# Evaluation of serum cancer antigen 125, resistin, leptin, homocysteine, and total antioxidant capacity in rat model of endometriosis treated with Curcumin

**DOI:** 10.14814/phy2.14016

**Published:** 2019-02-21

**Authors:** Gholamali Jelodar, Azimeh Azimifar

**Affiliations:** ^1^ Department of Basic Science School of Veterinary Medicine Shiraz University Shiraz Iran

**Keywords:** Curcumin, endometriosis, homocysteine, leptin, resistin, TAC

## Abstract

Endometriosis is one of the most common reproductive diseases of women, with some important biochemical changes in the serum. Curcumin was reported to have anti‐endometriosis, anti‐inflammatory and antioxidant activity. This study explores the changes of serum levels of Cancer Antigen 125 (CA125), leptin, resistin, homocysteine, and total antioxidant capacity (TAC) in a rat model of endometriosis and the effect of curcumin treatment on these factors. Fifty female Sprague‐Dawley rats (220–250 gr body weight) were randomly divided into control (received 0.3 mL of the vehicle), sham(stress of surgery + 0.3 mL of the vehicle) and three endometriosis groups as danazol treatment (7.2 mg/kg BW, IP), curcumin treatment (48 mg/kg BW, IP) and test (received 0.3 mL of the vehicle). Endometriosis was induced by surgically auto‐transplantation of uterine tissue to the abdominal wall and intestinal mesentery. The animals were treated for 4 weeks. On the last day, the blood sample was collected by heart puncture, and the above‐mentioned factors were measured in the sera. Leptin in the curcumin treatment group was markedly higher than all of the studied groups, except the danazol group, while there were no significant differences between other groups (*P* < 0.05). Level of resistin in endometriosis groups did not show significant differences with the control group (*P* < 0.05). There was no significant difference in the serum levels of homocysteine and CA 125 between all of the studied groups (*P* > 0.05). The serum levels of TAC in the control group were significantly higher than all of the studied groups (*P* < 0.05). Curcumin could prevent the growth of endometriosis, while there was no significant alteration on measured factors in the serum of rat with induced endometriosis. Hence follow up of these parameters in serum may not be a proper indicator to evaluate the status of endometriosis in the rat model.

## Introduction

One of the most common chronic, estrogen‐dependent illnesses of the female in reproductive age is endometriosis, a multifactorial disease associated with a general inflammatory response in the peritoneal cavity (Van Langendonckt et al. 2002). This disorder is defined by ectopic implantation and growth of endometrial tissue, primarily on the pelvic peritoneum and ovaries (Leyland et al. 2010). After adhesion, endometrial cells proliferate and gradually invade the peritoneal tissue. Oxidative stress has been suggested to be a predisposing factor for the pathophysiology of the disease and an increase in oxidative stress in women with pelvic endometriosis has been reported(Van Langendonckt et al. 2002).

Leptin as a proinflammatory cytokine (La Cava 2017) and resistin as a cysteine‐rich protein hormone which is linked to the complex role of adipocyte‐derived hormones in the immune system and during inflammation (Al‐Suhaimi and Shehzad 2013) are released by the visceral adipose tissue as postoperative cytokines (Lingohr et al. 2016), and are suspected to be involved in acute and chronic inflammatory metabolic disorders (Anderson et al. 2007).

Furthermore, it has been proposed that leptin may have a pathophysiological role in endometriosis and that its angiogenic and mitogenic action may contribute to the development and progression of endometriotic implants (Wu et al. 2002). On the other hand increase in resistin in the peritoneal fluid of women with endometriosis (Yi et al. 2010) and its expression in the ectopic endometrium of patients with endometriosis has been reported and was proposed to have a fundamental role in the pathogenesis of endometriosis (Oh et al. 2017).

Homocysteine is a thiol‐containing amino acid and it has been accepted that endometriosis is associated with an inflammatory process, with increased levels of homocysteine in the blood and follicular fluid (Ebisch et al. 2006; Natadisastra 2016). The main complaint of patients with endometriosis is infertility (Jacoeb et al. 2013). An increase in homocysteine levels may lead to increased levels of free radicals that subsequently lead to damage the oocyte, giving a poor quality of oocytes, decrease embryo quality and reduction in pregnancy success (Van Langendonckt et al. 2002).

Free oxygen radicals have been implicated in the pathogenesis of various diseases, ranging from male infertility (Wagner et al. 2018) to autoimmune deficiency syndrome (Kannan 2006). Enzymatic defense against oxidative stress includes: superoxide dismutase (SOD), catalase, glutathione peroxidase (GSH‐Px), glutaredoxin and glutathione reductase (Agarwal et al. 2008), and the degree of antioxidant defense present is often expressed as total antioxidant capacity (TAC).

CA‐125 is considered to be made by endometrial and mesothelial cells and enters the circulation in responses to inflammation via the endothelial of capillaries. It has been reported that in the women with endometriosis symptom the level of CA 125 ≥30 units/mL is highly specific for a diagnosis of endometriosis, but CA125 <30 *μ*/mL is unable to rule out endometriosis (Hirsch et al. 2016).

Current approaches for the treatment of endometriosis involve pharmacological therapy and surgical removal of endometriotic lesions which is not only temporarily effective, but also associated with a high recurrence rate (Guo 2009). Moreover, recent studies could identify several phytochemical compounds, including curcumin, which regress endometriotic lesions under experimental conditions (Zhang et al. 2011). Curcumin is a polyphenolic monomer extract of turmeric, a Chinese herbal medicine which activates microcirculation, possesses various pharmacological activities including anti‐inflammatory, antioxidant, free‐radical scavenger, and antiproliferative components (Partadiredja et al. 2013). Both curcumin and the Chinese medicine formula with turmeric may significantly alleviate and improve the symptoms of endometriosis patients (Qinshu et al. 2012). In spite of therapeutic efficacy, it has poor bioavailability (Anand et al. 2007), which appears to be primarily because of poor absorption, rapid metabolism, and rapid systemic elimination. This study was aimed to explore the effects of curcumin on changes of serum levels of leptin, resistin, homocysteine, total antioxidant capacity, and CA125 as biomarkers in the serum of a rat model of endometriosis. Danazol, which produces an environment with high androgen and low estrogen level leading to the atrophy of endometriotic implants, was used as the control intervention (Selak et al. 2007).

## Materials and Methods

### Animal ethics

This research study was carried out under the approval of the state committee on animal ethics, Shiraz University, Shiraz, Iran. Also, we followed the recommendations of the European Council Directive (86/609/EC) of November 24, 1986, regarding the standards in the protection of animals used for experimental purposes.

### Experimental design

Fifty female Sprague Dawley rats (180 ± 20 g), were purchased from Comparative and Experimental Center of Medical Sciences Department of Shiraz Medical University. The animals were kept under controlled lighting (12/12 h light‐darkness) and temperature (20 ± 2°C) and had free access to food and water. Animals were randomly divided into five equal groups as follows:

Group 1: Control group (received vehicle, 0.3 ml of ethanol 50% for 4 weeks).
Group 2: Sham group, this group received vehicle similar to group 1, following the opening of the abdomen and manipulation of visceral organs.

In the next three groups, endometriosis was induced as will be explained later and treated for 28 days as:

Group 3: Danazol treatment (received 7.2 mg/kg BW, intraperitoneal (IP).
Group 4: Curcumin treatment (received 48 mg/kg BW, IP).
Group 5: Test group (received vehicle similar to group 1).

As Curcumin (Sigma Aldrich Co. 3050) has low solubility in water and does not dissolve in ether or chloroform, ethanol was suggested as better solvent (Zhang et al. 2011). Therefore, we used ethanol 50% to make curcumin into a suspension for IP injection in rats.

### Induction of endometriosis

The method of auto‐transplantation was used in the present research for induction of endometriosis. Reproductive cycles of animals were checked by vaginal smear and rats in estrus phase were selected. Anesthesia was induced in rats with ketamine (50 mg/kg body weight) and xylazine (6 mg/kg body weight). The abdominal cavity was opened and left horn of the uterus was separated and was cut in 3 to 5 (3–5 mm) equal pieces and grafted to the abdominal wall and small intestine mesentery (Pelch et al. 2012).

### Blood sampling and hormonal assay

In the last day of experiments (day 28th) blood samples were collected under light anesthesia through heart puncture, and their sera were kept at −20°C till the analysis of required parameters. Total antioxidant capacity was measured (by ZB‐TAC‐96A, Zellobio, Germany) according to the manufacturer's description. All other parameters were measured by a rat‐specific kit (Cristal day kit, China).

In order to evaluate the induction of endometriosis in the operated groups (groups: 3 to 5), following collecting of blood sample, gross features of grafts were observed and histological samples of graft prepared and were evaluated for the confirming of endometriosis whenever it was existed.

### Statistical analysis

The results were expressed as mean ± standard error of the mean (SEM). Package for Social Sciences (IBM SPSS 19.0 for Windows) was used. The results were analyzed using the one‐way analysis of variance (ANOVA) followed by post hoc Tukey's multiple comparison test for comparison between different treatment groups. A value of *P* < 0.05 was considered statistically significant.

## Results

**Figure 1:**
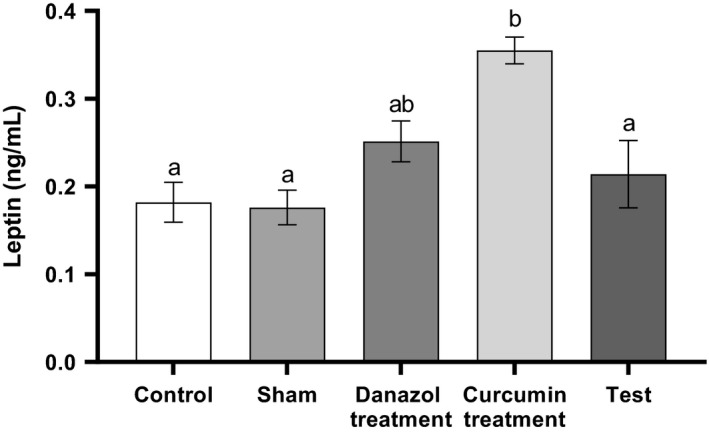
Serum levels of Leptin in different groups. Endometriosis was induced in Test, curcumin and Danazol groups. The analysis was performed using one‐way ANOVA followed by post hoc Tukey's multiple comparison test for comparison between different treatment groups (N = 10 in each group). Data are shown as mean ± SEM. A value of P < 0.05 was considered statistically significant. Groups with the similar alphabet on the top of the column are not significantly different, but different alphabets indicate a significant difference between groups, the label of “ab” means that it has no significant difference with columns labeled as “a” and “b” (P < 0.05).

Serum level of leptin in the curcumin treatment group was significantly higher than all of the study groups except the danazol treated group. There was no significant difference between the other groups (Fig. 1) (*P* > 0.05).

**Figure 2:**
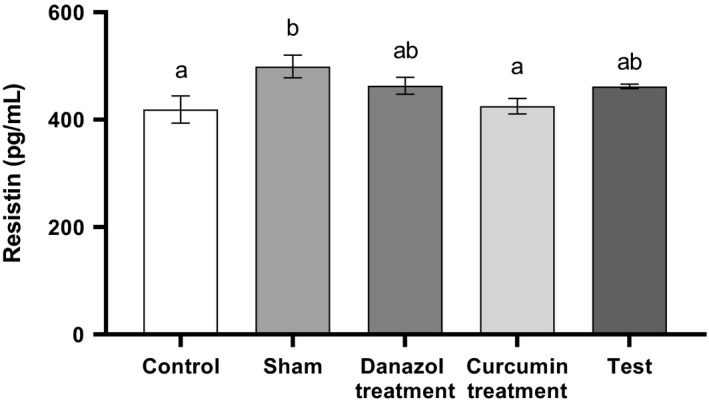
Comparison of Serum levels of resistin in different groups. Endometriosis was induced in Test, Curcumin and Danazol groups. The analysis was performed using one‐way ANOVA followed by post hoc Tukey's multiple comparison test for comparison between different treatment groups (N = 10 in each group). Data are shown as mean ± SEM. A value of P < 0.05 was considered statistically significant. Groups with the similar alphabet on the top of the column are not significantly different, but different alphabets indicate a significant difference between groups, the label of “ab” means that it has no significant difference with columns labeled as “a” and “b” (P < 0.05).

Although the level of resistin in the serum of the sham group was slightly but significantly higher than other groups, there was no significant difference between other study groups (Fig. 2) (*P* < 0.05).

**Figure 3:**
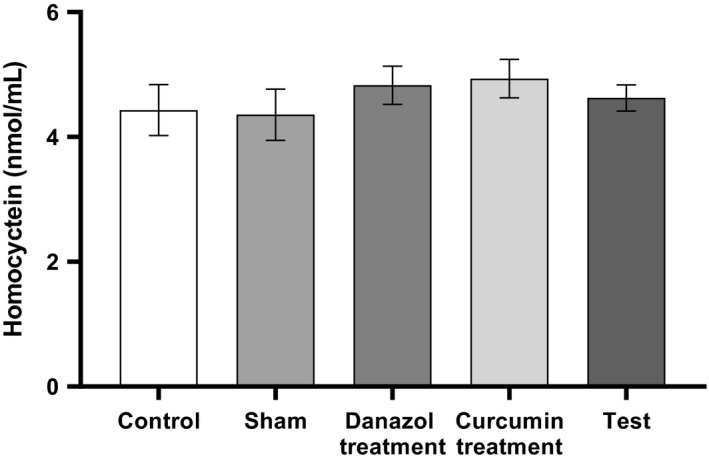
Comparison of Serum levels of homocysteine in different groups. Endometriosis was induced in Test, curcumin and Danazol groups. The analysis was performed using one‐way ANOVA followed by post hoc Tukey's multiple comparison test for comparison between different treatment groups (*N *=* *10 in each group). Data are shown as mean ± SEM. A value of *P* < 0.05 was considered statistically significant. There was no significant difference between groups (*P* > 0.05).

**Figure 4:**
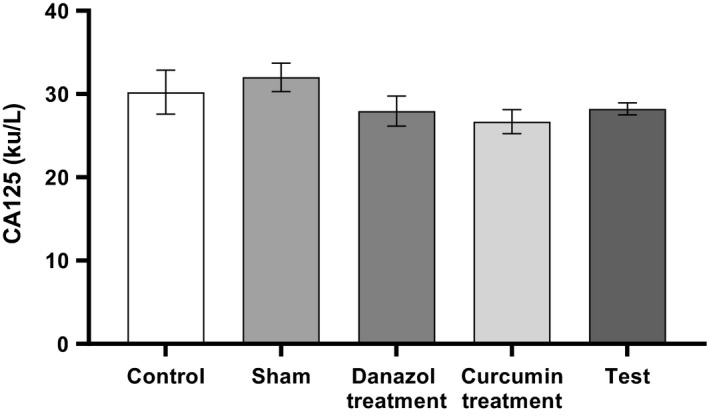
Comparison of Serum levels of CA125 in different groups. Endometriosis was induced in Test, curcumin and Danazol groups. The analysis was performed using one‐way ANOVA followed by post hoc Tukey's multiple comparison test for comparison between different treatment groups (N = 10 in each group). Data are shown as mean ± SEM. A value of P < 0.05 was considered statistically significant. There was no significant difference between groups (P > 0.05).

No significant difference in the serum levels of homocysteine and CA 125 noticed among the study groups (Figs. 3 and 4) (*P* > 0.05).

**Figure 5:**
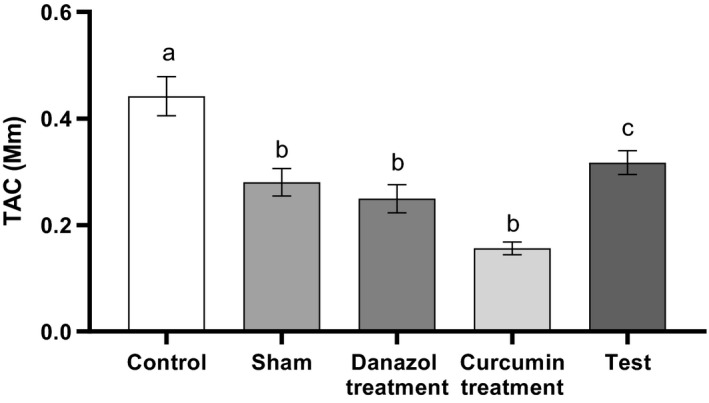
Comparison of Serum levels of TAC in different groups. Endometriosis was induced in Test, curcumin and Danazol groups. The analysis was performed using one‐way ANOVA followed by post hoc Tukey's multiple comparison test for comparison between different treatment groups (N = 10 in each group). Data are shown as mean ± SEM. A value of P < 0.05 was considered statistically significant. Groups with a similar alphabet on the top of the column are not significantly different, but different alphabets indicate a significant difference between groups (P < 0.05).

Serum level of TAC in the control group was significantly higher than all of the studied groups (*P* < 0.05). While in the curcumin treatment group it was markedly lower than other groups, it was only statistically significant compared to control and test groups (Fig. 5) (*P* > 0.05).

### Histopathological observation

**Figure 6:**
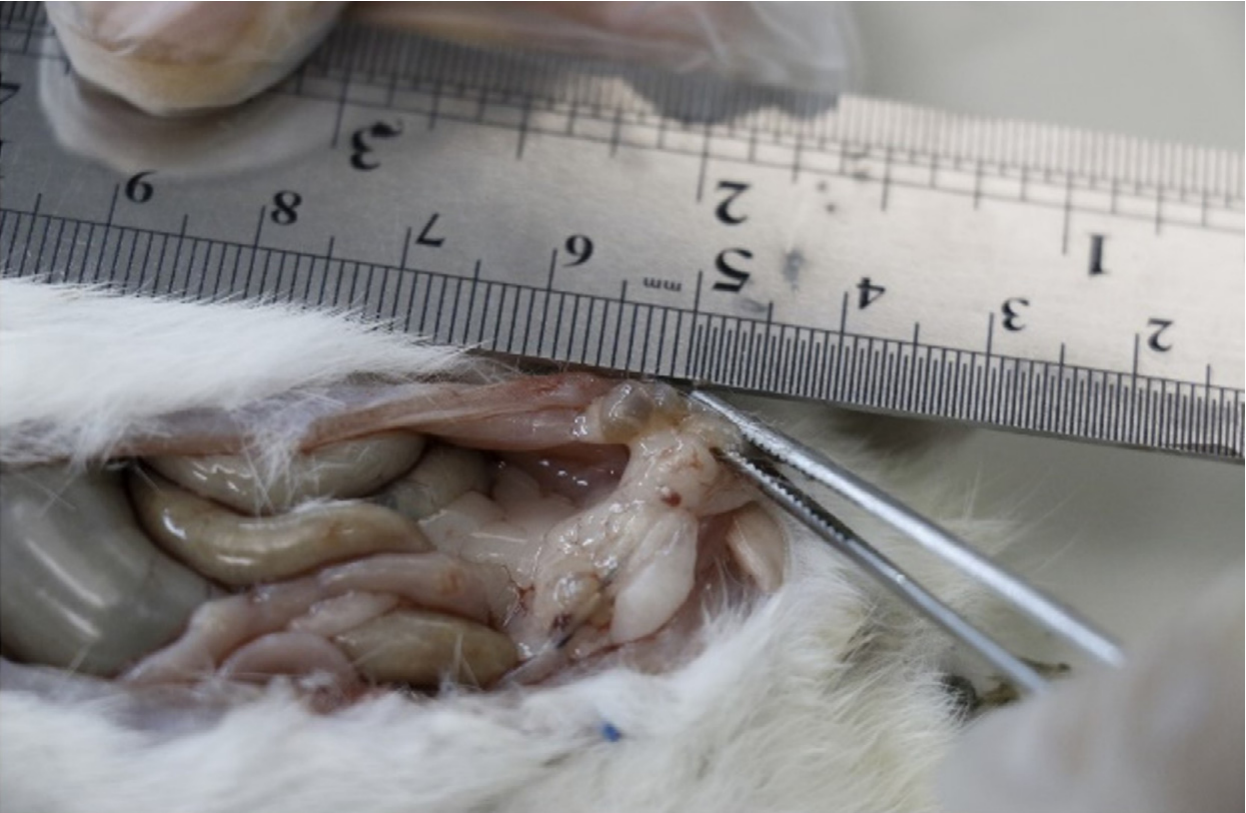
Endometrial lesions (endometriosis) on the abdominal wall, 4 weeks after Induction of endometriosis in the rat.

Following whole blood collection through the heart, the status of endometrial grafts in the experimental groups was grossly observed, measured, and histologically studied whenever it existed to confirm its relation to endometrial graft (Fig. 6). About 66.6% of grafts in the danazol treatment group existed and grew to a typical form of endometriosis, while no grown endometrial tissue was observed in the curcumin treatment group.

## Discussion

In this study, evaluation of serum level of leptin, resistin, homocysteine, CA125, and TAC was selected to find their possible alteration in an induced autograph model of endometriosis and following treatment with curcumin.

A significant difference was found in the serum level of leptin in the curcumin treatment group, but the level of leptin in other group remained unchanged.

While there was an attempt to offer leptin level as a biomarker for prediction of endometriosis (Seeber et al. 2008) contrasting reports exist on levels of leptin in endometriosis patients, showing no difference, an increase or a decrease. Markedly increased level of leptin mRNA and protein in rats with endometriosis was reported (Wu et al. 2002) but other reports did not support this proposal and concluded that serum leptin is unchanged in endometriosis (Gungor et al. 2009).

No significant alteration in the level of serum resistin noticed in endometriosis groups (treated & non‐treated) compare to the control group. Increased level of resistin in peritoneal fluid was proposed to be a powerful biomarker in the pathogenesis of endometriosis (Yi et al. 2010). Slight increase in resistin levels in the sham and endometriosis groups (nonsignificantly), which we have seen might be due to the inflammatory response to surgery (Asgeirsson et al. 2011). It should be considered that in our study resistin was measured in the serum, while its increase in the peritoneal fluid which has been reported is locally and could be in response to inflammation and metabolic disorder (Lehrke et al. 2004).

Endometriosis causes inflammation in the pelvic cavity and by interfering immune cell function and increasing the number of activated macrophages in the peritoneum, increases inflammatory mediators, such as homocysteine (Ebisch et al. 2006). It has been reported that the level of follicular fluid homocysteine increases in endometriosis (Jacoeb et al. 2013). However, in spite of our expectation, our results did not show a significant alteration in the serum level of homocysteine in all of the groups.

Level of TAC was significantly lower in the sham and endometriosis groups compared to the control group and it was the lowest in the curcumin‐treated group. As the anti‐inflammatory and antioxidant activities of curcumin were already reported (Siddiqui et al. 2006) it can be proposed that; decrease in TAC in our study is due to their usage to overcome the inflammation induced by endometriosis.

The initial report indicates that total antioxidant status is not altered in endometriosis (Polak et al. 1999). While others have shown that women with endometriosis‐associated infertility have an insufficient antioxidant defense, with lower TAC and significantly reduced SOD levels (Szczepanska et al. 2003; Lambrinoudaki et al. 2009).

Reactive Oxygen Species (ROS) was proposed as a factor which promotes the growth and adhesion of endometrial cells in the peritoneal cavity (Alpay et al. 2006) and oxidative stress play an important role in the development and progression of endometriosis (Harlev et al. 2015). However, some of the results failed to confirm an imbalance of ROS levels in the peritoneal fluid of patients with endometriosis (Bedaiwy et al. 2002), which may be explained by the fact that oxidative stress may occur locally only at the site of bleeding without affecting total peritoneal fluid ROS concentration (Agarwal et al. 2003). Moreover, changes of oxidative stress biomarkers may be transient and not be detectable at the time endometriosis diagnosis (Sekhon et al. 2010). Furthermore, the reason for these contradictory results can be differences in eligibility criteria, selection of control groups, and oxidative stress markers measured.

Level of CA125 did not show significant alteration among the groups, there is no strong evidence to support the alteration of this factor in experimental endometriosis. Moreover, there is controversy in reports regarding its changes in human endometriosis. While it was reported that evaluation of CA 125 may be useful for the diagnosis of deep endometriosis, especially when both samples are collected in midcycle and during menstruation (Oliveira et al. 2017) low level of CA 125 (<30 *μ*/mL) is however, unable to rule out endometriosis, and further investigation of alternative biomarkers to be both sensitive and specific for the diagnosis of endometriosis was recommended (Hirsch et al. 2016, 2017).

## Conclusion

Curcumin prevented the formation of the tumor while in danazol treatment group two third of grafts existed with tumors appearances. Although both danazol and curcumin with a preventive and therapeutic role in endometriosis to some extent suppressed the growth of endometriotic lesions, they could not exert a significant favorable change in our measured factors. In the control endometriosis group also changes of measured parameters did not show exactly, although these changes are not completely approved in human endometriosis, a similar pattern as what has been reported in human.

## Conflict of Interest

The authors report no conflict of interest. The authors alone are responsible for the content and writing of the paper.
